# Zeptomole Detection of C-Reactive Protein in Serum by a Nanoparticle Amplified Surface Plasmon Resonance Imaging Aptasensor

**DOI:** 10.1038/srep05129

**Published:** 2014-05-30

**Authors:** Stephen A. Vance, Marinella G. Sandros

**Affiliations:** 1 Department of Nanoscience, University of North Carolina at Greensboro, 2907 E. Lee Street, Greensboro, NC, USA 27401

## Abstract

Diagnostic biomarkers (i.e. proteins) are often in low abundance in bodily fluids presenting many challenges for their detection. In order to extend the application of SPRi systems in detecting biomarkers at ultralow levels, we combine the advantage of aptamer technology with nanomaterials and microwave-assisted surface functionalization. By implementing a sandwich assay through the introduction of aptamer-modified quantum dots (QDs), it was possible to measure 7 zeptomole (at 5 fg/mL) of C-reactive protein (CRP) selectively in spiked human serum. It is expected that the proposed platform will provide new direction in designing ultrasensitive SPRi biosensors with multiplexing capabilities.

An ultrasensitive platform with simultaneous fast profiling of multiple low abundance protein biomarkers from blood samples has the potential to provide a more comprehensive and accurate diagnosis/prognosis of different types of human diseases, including cancer, cardiovascular and neurological disorders. More importantly, a diagnostic biomarker should be able to highlight the early onset of a disease prior to the appearance of clinical symptoms to ensure a greater therapeutic efficacy. Based on these prerequisites, the detection platform should have super ultrasensitivity (below pg/mL), as biomarker levels in biological fluids are extremely low, to date, conventional platforms are unable to provide such ultrasensitivity in combination with multiplexing.

Enzyme linked immunosorbent assay (ELISA) is a commonly used technique for biomarker detection; it provides good sensitivity (pg/mL or femtomolar (10^−15^) range), excellent specificity, and low coefficient of variation (2–5%)^1^. However, the optimization of these assays is labor intensive, requires large amounts of sample in development and use, and can only target a single protein per assay. In addition, antibodies (recognition elements) in ELISA present practical limitations such as low stability and high production cost. An alternative recognition probe to antibodies are synthetic single stranded DNA/RNA aptamers which offer a large degree of specificity, high affinity, easy-to-perform modification, low cost and a rapid turnaround for production. Recently, aptamer-based biosensors exploiting detection by means of electrochemical^2^, optical^3^ and mass-sensitive transducers^4^,^5^ have been developed. Of these assay formats, surface plasmon resonance imaging (SPRi) is the most advanced label-free optical/mass-sensitive technology for detecting biomolecular interactions *in situ* and in real time with high throughput.

SPRi is an optical technique that monitors refractive index changes at the metal/dielectric media interface. A high refractive index prism coated with a thin layer of gold (50 nm) couples the incident light (p-polarized) to the propagating surface plasmons at a specific angle and wavelength^6^. Any perturbation to the sensor surface modifies the resonance conditions causing intensity variations of the reflected light at a fixed angle. The reflected light is then intercepted by a charge-coupled device (CCD) camera allowing for the visualization of multiple interactions simultaneously on the sensor chip in real time together with the relative sensorgram. The kinetic sensorgram plots the percent change in reflectivity versus time.

There have been significant advancements in SPR-based biosensors in the last two decades, however, measuring ultra-low levels (sub-ng/mL) of biomarkers in bodily fluids still remains a challenge. To this end, a number of amplification techniques combining proteomics^7^ and genomics^8^,^9^,^10^ with nanomaterials have been proposed to increase the signal, or optical contrast, generated by the binding event in SPR. For example, one strategy involves utilizing a sandwich-immunoassay type complex through the inclusion of gold nanoparticles in the sensing layer to detect biomarkers in serum^11^ and plasma^12^ with a detection limit range between 0.1–2.3 ng/mL. The SPR signal amplification is attributed to the coupling of localized surface plasmon resonance with SPR and a mass loading effect^13^. Other strategies take advantage of the coupling of fluorescent probes such as quantum dots (QDs) to metallic surfaces^14^,^15^,^16^. Plasmonic field effects are known to improve the emission from fluorescent probes by several fold to produce highly directional and polarized emissions^17^. Moreover, the refractive index near the plasmonic surface is modified because of the presence of the fluorescent probes, thus affecting the excitation and propagation of the plasmon wave^18^,^19^. Recently, we have reported that the integration of near-infrared (NIR) QDs with SPRi enhanced the limit of detection (LOD, pg/mL)^15^ due to a mass loading effect and spontaneous emission coupling with propagating surface plasmons.

In the present communication, we introduce an ultrasensitive SPRi-based nano-aptasensor for the detection of C-reactive protein (CRP) at 5 fg/mL (43 aM [10^−18^ M] and/or 7 zeptomole [10^−21^ moles, (assay volume of 150 μL)]) level in spiked human serum. This ultrasensitive system was engineered through the unique integration and combination of the SPRi platform with microwave-assisted surface chemistry, aptamer technology and NIR QDs, to create a clinically relevant biosensor. CRP is a general inflammatory biomarker and useful in diagnosing inflammatory responses in cancer^20^, cardiovascular diseases^21^ and neurological disorders^22^. In this study, CRP was simply employed as a model biomarker. To accurately diagnose most human diseases, panels of biomarkers need to be profiled simultaneously at real time with high sensitivity. Due to the innate capabilities of the SPRi instrument for multiplexing and the proposed nano-aptasensor attomolar sensitivity, one can foresee the potential of extending this proposed platform to detect in real-time proteinaceous and non-proteinaceous molecules found in blood, urine, cerebrospinal fluid and other specimens that are indicative and even predictive of disease onset and progression.

## Results

### Microwave-assisted surface functionalization

**Figure 1:**
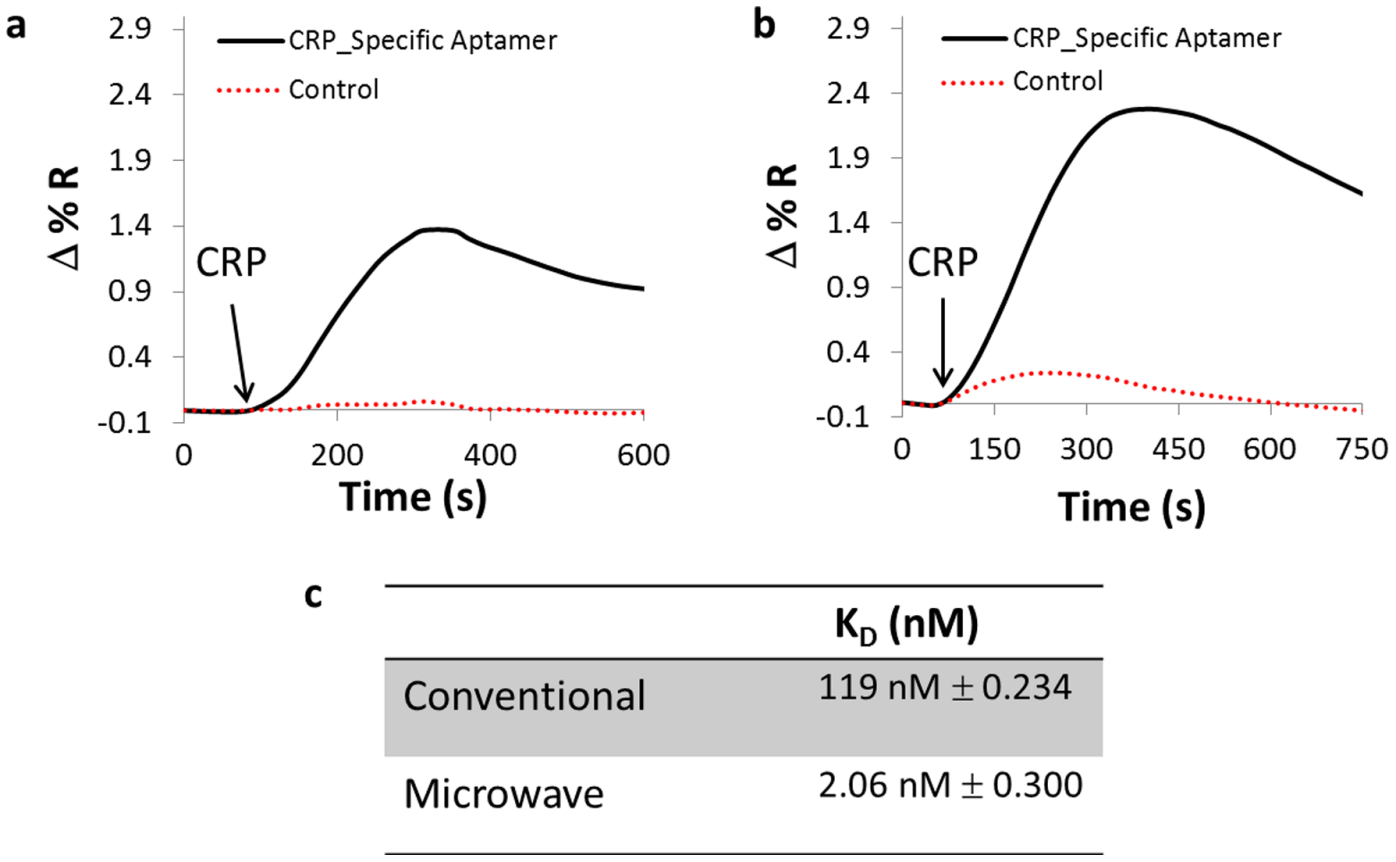
Binding of CRP to sensor chip using conventional or microwave-assisted surface functionalization. A plot comparsion of the SPRi kinetic signal after the injection of CRP (2 μg/ml) in buffer (10 mM TRIS, 15 mM NaCl, 2 mM CaCl2 pH 7.4) onto a gold-coated prism that has been coated with Cystamine/Glutaraldehyde in the (a) absence and (b) the presence of microwave-assisted irradiation and followed by the functionalization of aminated CRP-specific and control aptamer. Both surfaces were blocked with BSA prior to injection of CRP. (c) A comparison of the equilibrium dissociation constant (K_D_) between microwave and conventional treated surface to the binding response of CRP.

In general, for a SPRi biosensor, the functionalization of chemical linkers on the surface of the gold-coated chip serves to provide means of attaching the capture probe. Conventional immobilization procedures using Cystamine/Glutaraldehyde (Cys/Glu) layers requires over 3 hours prior to introduction of the capture probe. In this study, we compared conventional methods with microwave-assisted surface functionalization of Cys/Glu to assess their influence on the binding interaction between the capture and target probe. In all SPRi experiments, the change in reflectivity (%ΔR) was calculated by taking the difference between the initial and final buffer signals. As Fig. 1 shows the microwave treated chip had a significantly larger signal change (% Δ R, 2 fold increase) after injection of CRP (a model biomarker) than the chip that was prepared through the conventional method. The calculated equilibrium dissociation constant (K_D_, Fig. 1c) of the CRP aptamer decreased two order of magnitudes resulting in a stronger binding interaction. Microwave-assisted surface functionalization not only improved the detection of CRP and avidity using SPRi but also decreased the functionalization procedure from several hours to minutes.

### Blocking agent influence and optimization

**Figure 2:**
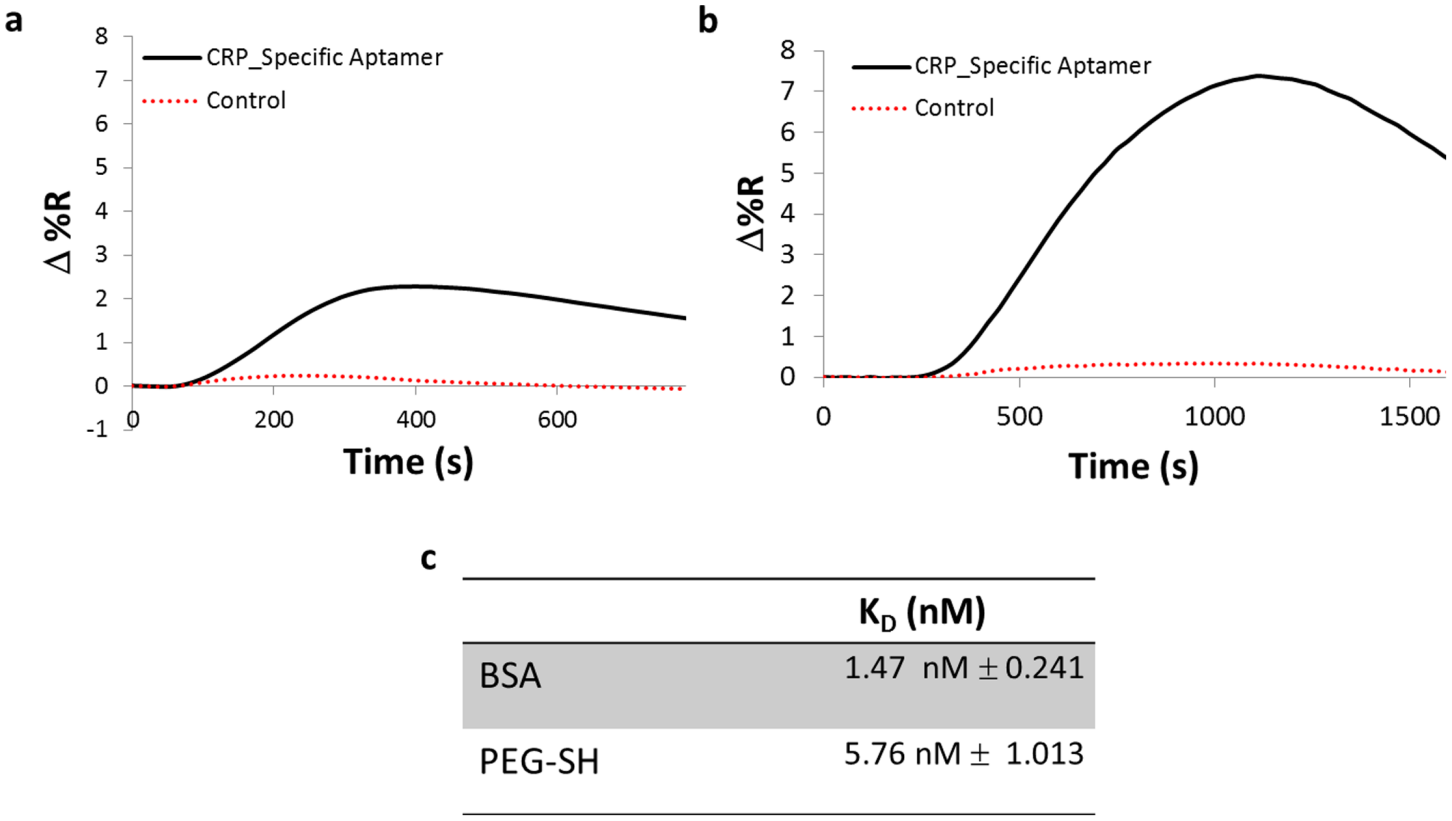
CRP binding to sensor chip blocked with BSA or PEG-SH. A plot comparison of the SPRi kinetic signal after the injection of CRP (2 μg/ml) in buffer (10 mM TRIS, 15 mM NaCl, 2 mM CaCl2 pH 7.4) onto a gold-coated prism that has been functionalized with biotinylated CRP-specific and control aptamer followed by blocking with (a) BSA and (b) PEG-SH. (c) A comparison of the equilibrium dissociation constant (K_D_) between BSA and PEG-SH treated surface to the binding response of CRP.

In addition to the immobilization of a chemical linker directly onto the gold surface for subsequent attachment of probes, the overall goal is to engineer a support construct that provides large degree of accessibility to the target probe while retaining good stability and minimizing capture probe detachment and non-specific binding. Extravidin was selected as the direct chemical linker to the capture probe in order to take advantage of its strong binding affinity to biotinylated probes, as well as its superior antifouling properties against human serum proteins. After the formation of the Cys/Glu layer, the gold surface was blocked with chemicals to prevent any non-specific adsorption from serum proteins. To this end, we compared two commonly used blocking agents, bovine serum albumin (BSA) and poly(ethylene glycol) methyl ether thiol (PEG-SH). After direct binding of the capture probe (biotinylated CRP_Specific Aptamer) to an extravidin coated surface, followed by injection of CRP in buffer, the PEG-SH had a greater SPRi response than BSA (Fig. 2a–b). When comparing their binding affinity both exhibited similar values (Fig. 2c), however, PEG-SH provided better accessibility for CRP to bind to the immobilized aptamer.

### Determination of SPRi limit of detection for CRP in human serum

**Figure 3:**
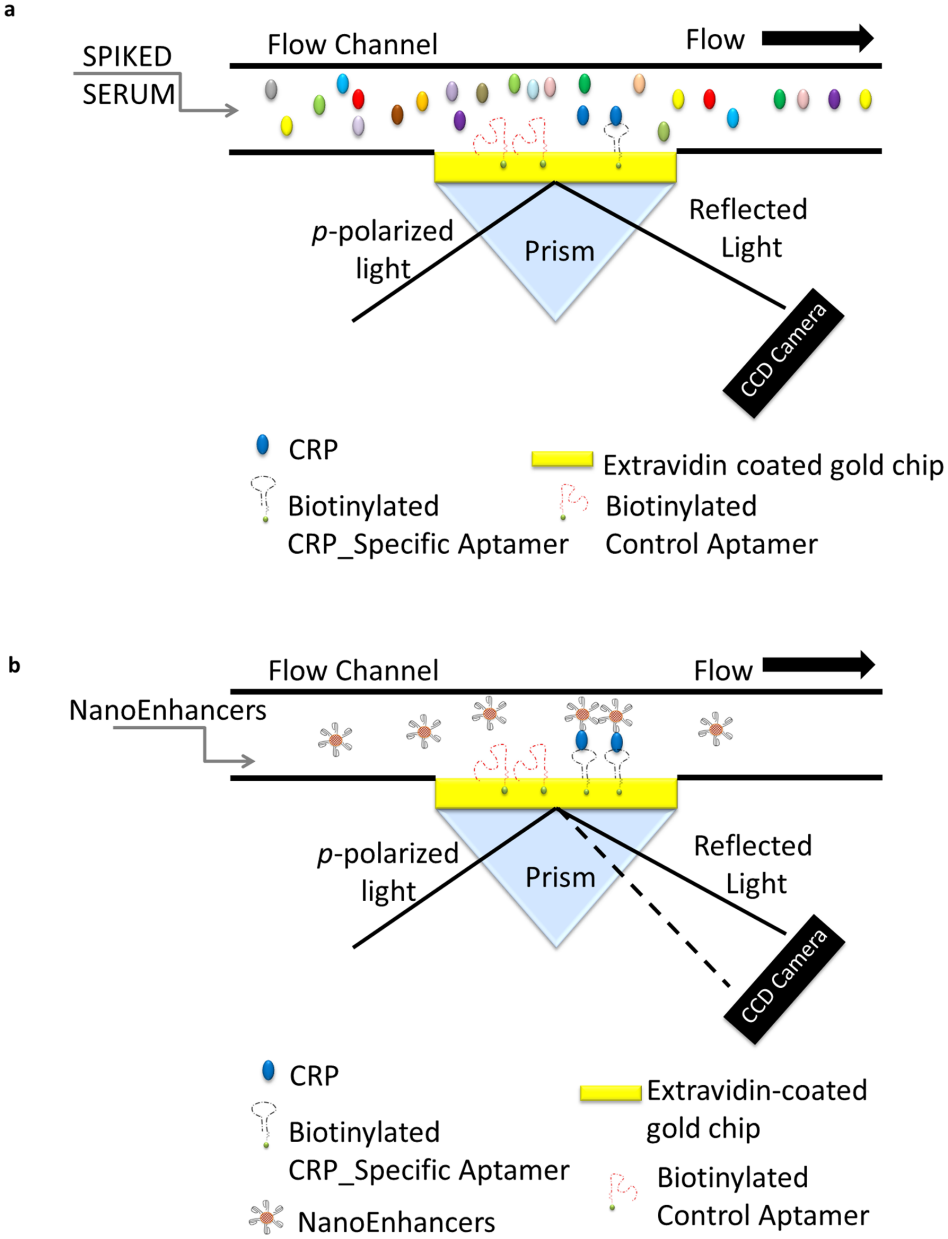
A schematic illustration of the sandwich protocol implemented for the detection of CRP in biological fluid. The gold-coated prism is pre-functionalized with aptamers specific to CRP and control aptamers followed by the (a) direct detection of CRP (fg/ml) spiked in human serum and the (b) sandwich based assay using CRP-Specific_aptamer-coated QDs for SPRi signal amplification. Direct detection of CRP (fg/ml) does not generate a quantifiable sensor response as depicted with no change in the angle of reflectivity, however, with sandwich assay the NanoEnhancers induce a change in the reflectivity.

Our final construct for the detection platform involved using a microwave-assisted Cys/Glu coated gold chip that was immobilized with extravidin for direct attachment of the capture probe (biotinylate CRP_specific aptamer). The sensor chip was further blocked by injecting PEG-SH inside the instrument. Following the injection of a solution of CRP spiked in 1% human serum, an intense SPR signal response with a weak association and shortly after an abrupt signal drop (Supplementary Fig. S1) were observed. The intense SPR response is due to the high refractive index difference between the running buffer and the serum. In addition, it could be attributed to weak non-specific interaction of serum proteins. Therefore, using the direct detection method for CRP spiked in human serum is challenging, as one cannot precisely quantify the amount of CRP (Fig. 3a).

**Figure 4:**
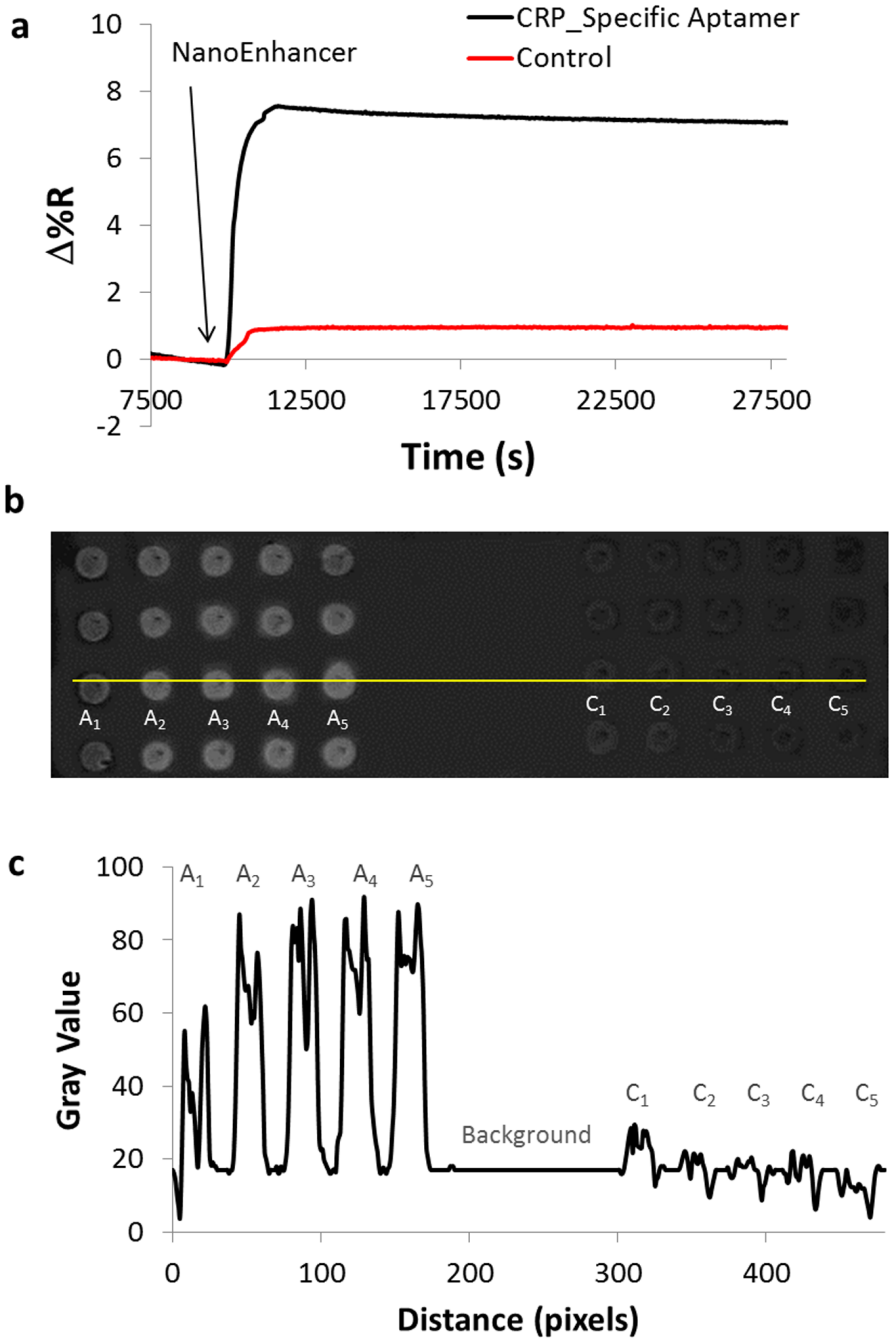
Detection of CRP using a sandwich assay spiked in human serum. (a) Binding of NanoEnhancers (CRP_specific_Aptamer-QDs) after the injection of PEG-SH and CRP (500 pg/ml) spiked in human serum to Cys/Glu/extravidin/Aptamer surface coated gold chip and (b) SPRi difference images showing the binding of NanoEnhancers to CRP (left) and control (right). (c) A plot profile of the SPRi difference image revealing intensity values to the area indicated by the yellow line in figure 4b and shows the change in contrast due to the binding of NanoEnhancers in spots functionalized with CRP_Specific aptamer (left, A1–A5), control aptamer (right, C1–C5). The middle region of the line is the background.

To overcome this challenge, we designed a sandwich based assay that uses CRP_specific Aptamer coated NIR QDs (NanoEnhancers) to amplify the signal for the SPRi sensor (Fig. 3b). As shown in Fig. 4a, the introduction of the NanoEnhancers to a pre-injected solution of CRP (500 ng/mL) in human serum resulted in 17.85 ± 0.54 (%ΔR), while the control sample had minimal change (%ΔR = 1.74 ± 0.22). In addition, a difference image (Fig. 4b) was recorded to correlate the binding kinetics after injection of NanoEnhancers. The spots that have been pre-functionalized with CRP_specific Aptamers (left) are intensely illuminated as opposed to control aptamers spots (right). The correlation between the binding kinetics and the difference images (plot profile) helps further validate the binding interaction between aptamer-CRP and NanoEnhancers. The formation of the NanoEnhancers-CRP complex (K_D_ = 169 pM ± 1.6) resulted in higher affinity in comparison to the formation of Aptamer-CRP complex (K_D_ = 5.76 nM ± 1.01). Furthermore, we examined the influence of BSA in the sandwich based assay format and found that there was no signal enhancement after the addition of the nanoEnhancers (Supplementary Fig. S2).

**Figure 5:**
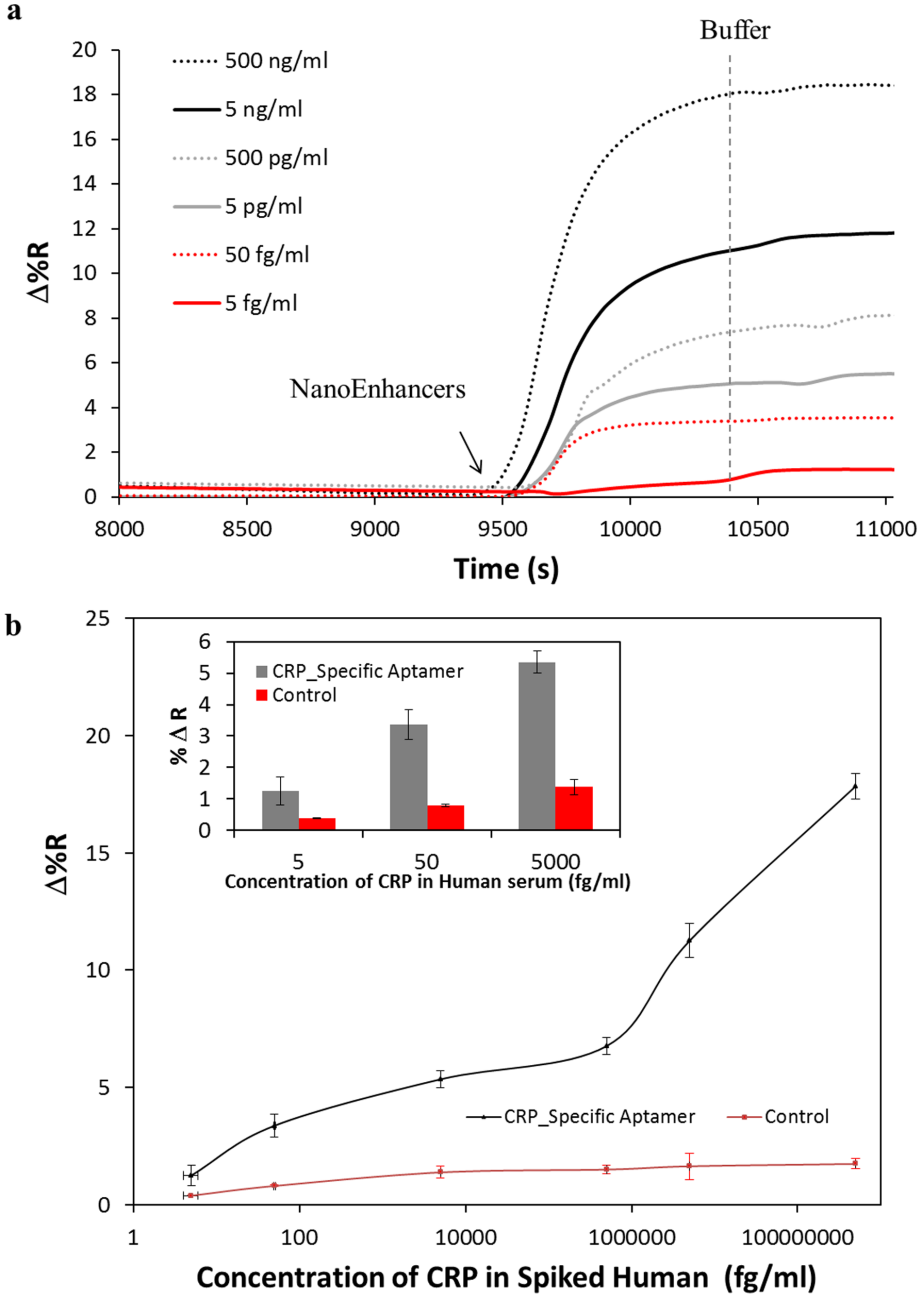
A sandwich assay using NanoEnhancers for the detection of CRP spiked in human serum. (a) Normalized SPRi kinetic plot representation of CRP_specific_Aptamer-QDs-amplified signal for human serum samples spiked with different concentrations of CRP. A vertical dashed line (grey) represents the injection point of the running buffer. (b) A concentration gradient curve representing the binding of NanoEnhancers (CRP_specific_Aptamer-QDs) after the injection of various amounts of CRP spiked in human serum to the sensor surface that has been prefunctionalized with biotinylated CRP-specific (black) and random aptamer as a control (red). The inset figure depicts the percent change in reflectivity (%ΔR) after introduction of NanoEnhancers for 5 fg/ml, 50 fg/ml and 5000 fg/ml.

To further investigate the reliability of the biosensor in distinguishing different amounts of CRP present in human serum, we assessed the sensor performance with a range of CRP concentrations. A decrease in SPRi signal was observed as the amount of CRP spiked in human serum was lowered (Fig. 5a). To further assess the robustness of our platform, serum concentration was increased from 1% to 20%. A complementary response was observed after the injection of 500 pg/ml of CRP in 20% serum (Supplementary Fig. S3) in comparison to the corresponding experiment performed in 1% serum (Fig. 5a). Examining the extended range of the concentration gradient curve in Figure 5b, the detection platform exhibited a biphasic response that can be interpreted as the result of two binding sites with different affinities. The biphasic response is common with SPR sensors when proteins multimerize. In our case, CRP in the presence of calcium, conforms to a pentameric structure^23^, as a result, two binding sites with different affinities are observed. However, at the lower concentration range, the detection platform exhibited a linear trend in the region from 5 fg/mL to 5000 fg/mL (Supplementary Fig. S4). The LOD was validated to be 5 fg/mL, this in agreement with the LOD having a 3-fold higher response than the control, when taken into consideration the standard error (inset, Fig. 5b).

## Discussion

Microwave assisted chip-surface modification offers fast turnaround time and a stronger support structure for the capture probe. The acceleration in reaction time with microwave irradiation^24^, in comparison to conventional methods, is due to the conversion of electromagnetic energy into heat, rendering a robust SPRi sensor with better sensitivity and avidity. In addition, the aptamer binding affinity was comparable to CRP antibodies^25^. We have found that the type of blocking agent used greatly influences the capture probe interaction with analyte, as well as with the NanoEnhancers. For potential clinical applications of SPRi, much effort was spent in optimizing the surface chemistry of the sensor; the combination of PEG-SH/Extravidin^26^ and microwave-assisted functionalization with Cys/Glu was found to significantly improve the efficiency and performance of the biosensor.

There are several challenges in directly detecting biomarkers in human serum using the SPRi platform, because the direct response from the binding of biomarker to the sensor surface can be masked by non-specific interactions from sera proteins (higher in concentration than desired biomarker), changes in the refractive index of injected solution from the running buffer, and analyte concentration falling below the LOD of the instrument. To overcome these obstacles, we employed a sandwich-amplification strategy using NanoEnhancers that allowed us to detect CRP (model biomarker) in human serum at a LOD of 43 attomolar (5 fg/mL). Many previously reported strategies utilize gold nanoparticles in complex samples^11^,^12^,^27^ or buffered conditions^18^,^28^,^29^ to amplify the SPR signal for detection of biomolecules; however, the level of sensitivity attained with our platform is by far superior. Previously, we reported the use of NIR QDs for signal amplification of prostate specific antigen (PSA) using antibodies as capture probes and PEG-COOH/PEG-OH as the surface coating and attained a LOD of 2.5 ng/mL. In the present work, we employed aptamers as opposed to antibodies^15^ to serve as the capture probe. We have found that by using aptamers in a sandwich assay, sensitivity of the sensor is improved, as observed by Kim and co-workers^4^. We attribute this increase in sensitivity to a decrease in the separation distance between the QDs and the sensor surface, as a result of the aptamer folding from a single stranded DNA to tertiary structure after binding to analyte, or perhaps due to differences in length between aptamer and antibody. In addition, we observed that the aptamers conjugated on quantum dots (NanoEnhancers) experienced a one order of magnitude improvement in affinity compared to its parent aptamer (immobilized capture molecule). This enhancement could be a result of several aptamers and CRP binding^30^. Increasing the serum concentration from 1% to 20% did not affect the percent change in reflectivity after the addition of NanoEnhancers. However, to minimize non-specific binding from excess proteins present in the sample, the salt concentration in the running buffer was increased from 15 mM to 250 mM.

The exact mechanism behind NIR QDs SPRi signal enhancement is still not very well understood, however, few hypotheses could be presented here. One hypothesis alludes to a mass loading effect and the other suggests NIR fluorophores will couple the scatter light more strongly onto gold film nanostructures^31^. Nanometer thick gold film have a stronger absorption^32^ in the NIR as opposed to the visible range. As highlighted in our previous work^15^, the visible red emitting quantum dots (550–650 nm) had a lower SPR amplification signal than the NIR quantum dots (800 nm). Furthermore, Wei et al.^19^ refers to a bidirectional relationship between QDs and SPs on a silver nanowire, where energy is transferred from propagating SPs to the excitons and the excited QDs prompt the generation of propagating surface plasmons in the silver nanowires. All above mentioned work suggest that metallic film-QD heterostructures experience unique interactions that we are still in the process of exploring greatly to further understand their principal mechanism and applications. Finally, this work highlights and establishes the ability of SPRi sensors to achieve zeptomole sensitivity in biological fluids as a result of combining NIR QDs with smart surface engineering.

## Methods

### Chemicals and reagents

Cystamine dihydrochloride (Cys), glutaraldehyde solution 25% (Glu), phosphate buffered saline (PBS), poly(ethylene glycol) methyl ether thiol (PEG-SH), bovine serum albumin (BSA), extravidin were purchased from Sigma-Aldrich(St. Louis, MO, U.S.A.). Calcium chloride anhydrous (CaCl_2_), sodium chloride (NaCl), TRIS base for molecular biology and ethanol were all purchased from Fischer Scientific. Nanostrip was purchased from Cyantek. Biotinylated CRP_specific Aptamer was purchased from OTC Biotech. Biotinylated control aptamer with the following sequence 5′-GGGCCTCCGGT -TCATGCCGC-3′ was purchased from Integrated DNA technologies. QDot 800 streptavidin conjugate was purchased from Life Technologies.

### SPRi gold chip cleaning

The gold-coated prism (Horiba Scientific, France) was sonicated in water for 30 minutes at 50°C and then rinsed with ethanol and dried in the oven (60°C). To remove any organic contaminants, the gold-coated prism was then immersed in nanostrip and then heated to 50°C under sonication for 90 minutes. The solution was then allowed to cool to room temperature and then the prism was removed and rinsed with water to remove excess Nanostrip followed by sonication in water (50°C) for 30 minutes. The prism was then given a final wash with ethanol and dried with nitrogen. Finally, the prism was then placed into a UV Ozone Cleaner (ProCleaner™ Plus from Bioforce Nanosciences) for 20 minutes. If it is not noted below after the formation of each layer, the biochip was excessively rinsed with water and then dried with nitrogen.

### Conventional surface functionalization

To bind biotin-labeled aptamers onto the gold surface, we used Cystamine/Glutaraldehyde/Extravidin surface chemistry. In Brief, the biochip was immersed in 25 mM cystamine in 90% ethanol for 2 hrs and washed with ethanol. Afterwards, a second layer was formed by dipping the chip in a 2.5% solution of glutaraldehyde for 1 hr. Finally, extravidin (0.2 mg/mL diluted in PBS, pH 7.4) was deposited on the chip and incubated for one hour in a humid environment and rinsed with water followed by drying with nitrogen. Biotin-labeled CRP and control aptamers (10 μM) suspended in 10% glycerol were spotted (300 μm) using a SPRi Arrayer (Horiba Scientific) onto the surface and left to incubate for 2 hours at a humidity of at least 75%.

### Microwave-assisted surface functionalization

A cleaned gold-coated chip was immersed in a solution of cystamine (25 mM in 90% ethanol) and then microwave irradiated (50 watts, 5 minutes, 50°C) using a CEM discover Labmate. Afterwards, the prism was rinsed and soaked for 5 minutes in 90% ethanol. The second layer formation involved depositing the chip in a 2.5% glutaraldehyde solution and irradiated with the same microwave settings as described with cystamine. A solution of extravidin (0.2 mg/mL diluted in PBS buffer, pH 7.4) was deposited on the chip surface. Spotting of the biotinylated aptamers was done in the same manner as described in the conventional surface functionalization section above.

### SPRi measurements

SPRi measurements were performed using SPRi Lab+ instrument equipped with an 800 nm laser, CCD camera, peek flow cell, programmable syringe pump (Harvard Apparatus PHD 2000) and an injection loop of 150 μl (Horiba Scientific, France) placed in a Memmert Peltier-cooled incubator (model IPP 500, Wisconsin Oven Distributors, USA) for temperature stabilization. The entire prism surface was monitored during the experiments; spots with a diameter of 300 μm were chosen to determine total reflectance change that indicates a binding event occurrence. For each injection, 40 SPRi signals were collected, background subtracted automatically and averaged using the ScrubberGen Software. Each kinetic curve corresponds to an average curve of 20 spots for CRP-Specific Aptamer and 20 spots for the control. The reproducibility of each measurement was thus expressed as percent change in reflectivity on the 40 SPRi signals relative to each sample injected on four different biochips (n = 4). Reflectance change was monitored at the angle that is determined to be the highest slope of the plasmon absorption. The kinetics analysis was performed by plotting percent change in reflectivity (%ΔR) against time to illustrate the binding events. The SPRi difference images taken by the CCD camera were collected at real time to monitor the reactions occurring on the surface of the chip. The binding event was observed as an increase in the reflected intensity, regarded as a bright spot, which is easily distinguishable from the background (black). The reported curves are the average of 20 spots (background = blocked surface reflectance change subtracted), each experiment has been repeated four times. The difference images were then used to show the reflectivity change to further confirm that a binding event has occurred. To retain the pentameric structure of CRP, the running buffer contained calcium. Protein binding experiments were performed at a flow rate of 5 μl/min using a running buffer of 10 mM Tris, 15 or 250 mM NaCl (the former concentration was used in 1% serum experiment and the latter in 20% serum experiment), 2 mM CaCl_2_ at pH 7.4. The prism was then blocked with an injection of 10 mM PEG-SH or 1% BSA followed by a running buffer rinse. The SPRi was then calibrated by injecting a sample of the running buffer that has a 25 or 260 mM NaCl concentration instead of the normal 15 or 250 mM. This injection causes an increase in reflectivity due to the change in the dielectric constant. A calibration factor for each plot was then calculated and used to adjust all of the plots to the same change in reflectivity. CRP in running buffer or spiked in 1% or 20% human serum was then injected. For all sandwich assays, 10 nM of Streptavidin Qdot 800 was reacted with biotinylated CRP_Specific Aptamer (NanoEnhancers) for 30 minutes prior to injection. The NanoEnhancers were diluted with running buffer prior to injection into the SPRi system. Finally, %ΔR was computed by taking the difference between pre and post NanoEnhancers or protein injection (initial and final buffer signals). The reported limit of detection (LOD) represents the minimum detectable target concentration for which the SPRi signal (%ΔR) was at least three times higher than that of the control. Equilibrium dissociation constants (K_D_) were calculated using the ScrubberGen software.

## Author Contributions

M.G.S. conceived and designed the experiments. S.A.V. carried out the experiments. M.G.S. and S.A.V. wrote the paper. All the authors discussed the results and commented on the manuscript.

## Supplementary Material

Supplementary Information Supplementray Information
