# Supplementary material for: Zeptomole Detection of C-Reactive Protein in Serum by a Nanoparticle Amplified Surface Plasmon Resonance Imaging Aptasensor
Source: Sci Rep. 2014 May 30;4:5129. doi: 10.1038/srep05129 (PMC4038818; doi:10.1038/srep05129)
Supplement: Supplementary Information — Supplementray Information [file srep05129-s1.pdf]

Supplementary Information

Zeptomole Detection of C-Reactive Protein in Serum by a Nanoparticle Amplified Surface Plasmon Resonance Imaging Aptasensor

Stephen A. Vance & Marinella G. Sandros*

Department of Nanoscience, University of North Carolina at Greensboro, 2907 E. Lee Street, Greensboro, NC, USA 27401

*Address Correspondence to: m_sandro@uncg.edu

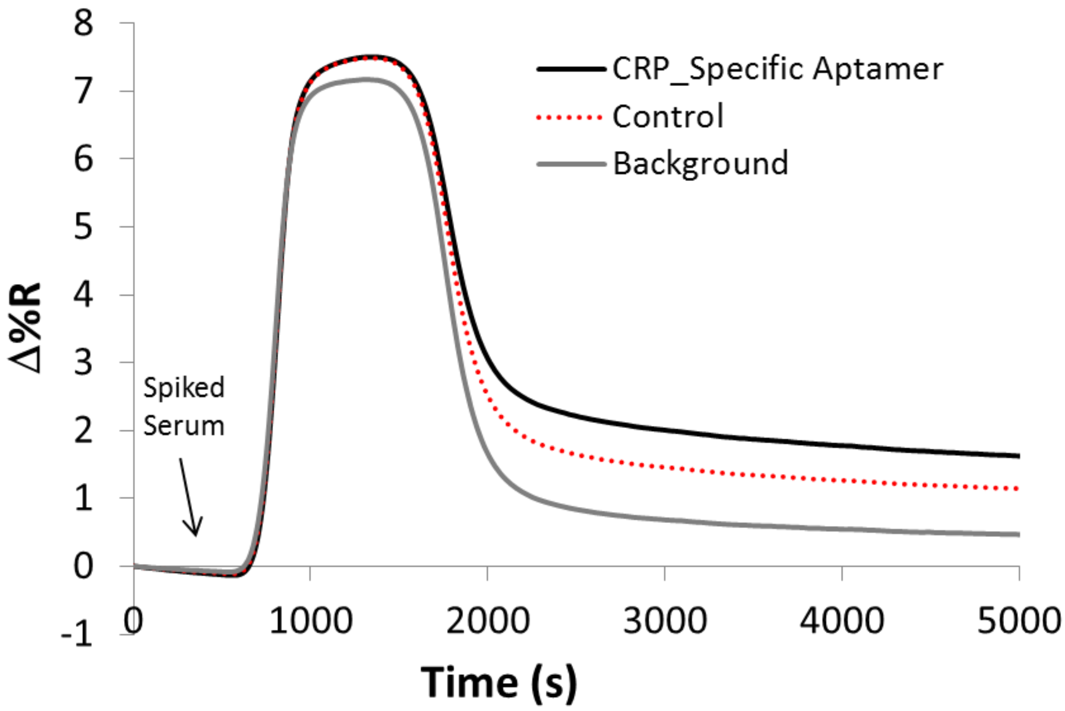

Supplementary Figure S1| SPRi sensor response after the injection of CRP spiked in serum. A SPRi kinetic plot after the injection of PEG-SH followed by CRP (500 ng/ml) spiked in human serum to a pre-functionalized chip with Cys/Glu/Extravidin/biotinylated CRP-specific Aptamer and control aptamer.

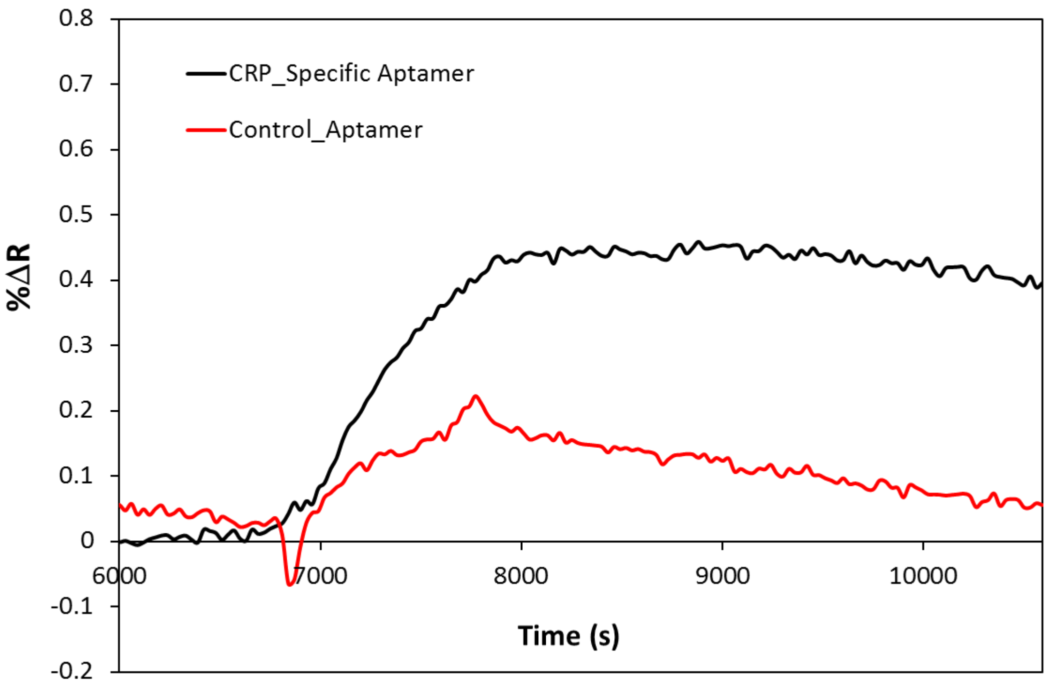

Supplementary Figure S2| SPRi sensor response to blocking with BSA. Introduction of nanoEnhancers (CRP_specific_Aptamer-QDs) after the injection of BSA followed by CRP (500 ng/ml) spiked in human serum to Cys/Glu/extravidin/Aptamer surface coated gold chip.

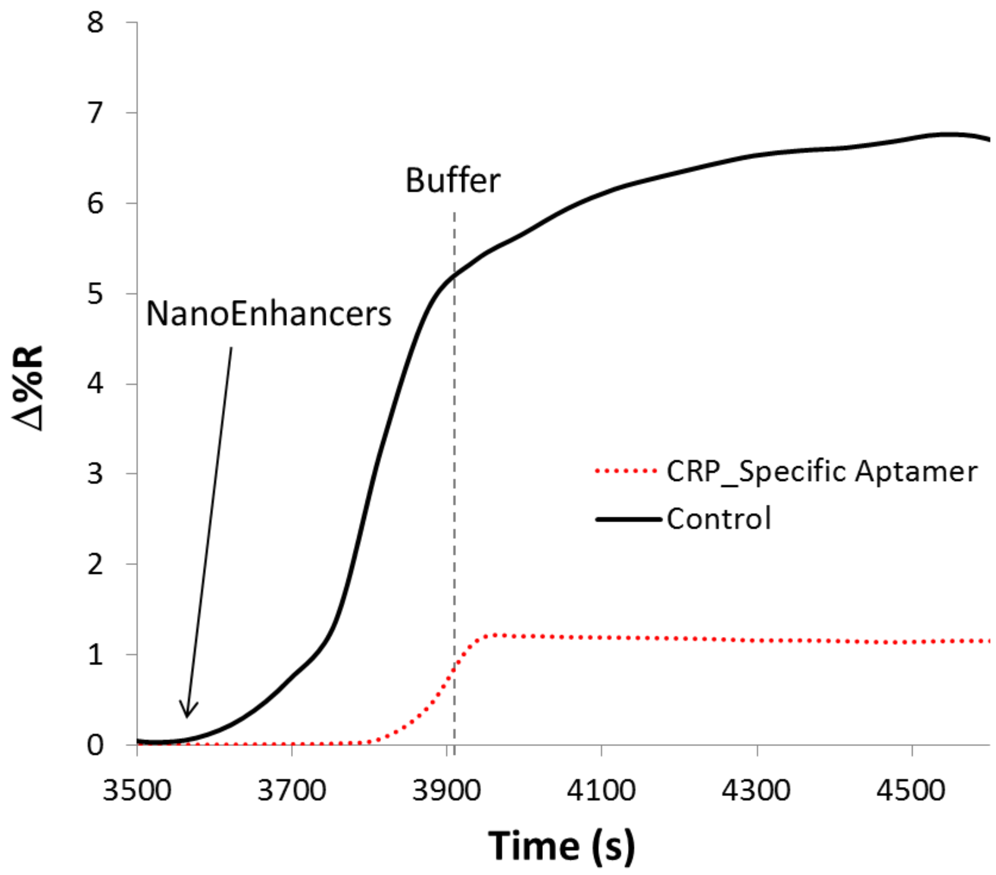

Supplementary Figure S3| Detection of CRP using a sandwich assay spiked in 20 % human serum. (a) Binding of NanoEnhancers (CRP_specific_Aptamer-QDs) after the injection of PEG-SH and CRP (500 pg/ml) spiked in human serum to Cys/Glu/extravidin/Aptamer surface coated gold chip. A vertical dashed line (grey) represents injection point of running buffer.

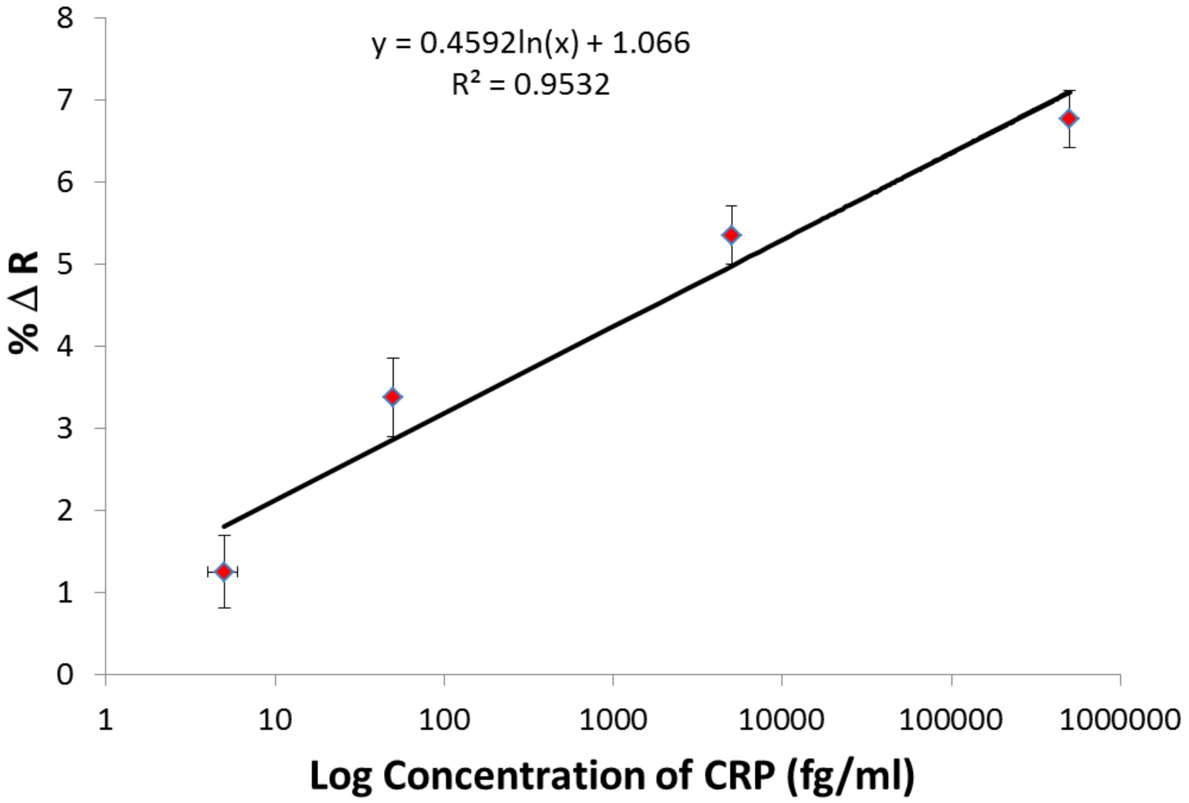

Supplementary Figure S4| A calibration curve for CRP by using a sandwich assay. Linearity of SPRi response against the CRP concentration spiked in human serum after the injection of NanoEnhancers for signal amplification.
